# Multifractal Analysis for Nutritional Assessment

**DOI:** 10.1371/journal.pone.0069000

**Published:** 2013-08-21

**Authors:** Youngja Park, Kichun Lee, Thomas R. Ziegler, Greg S. Martin, Gautam Hebbar, Brani Vidakovic, Dean P. Jones

**Affiliations:** 1 Division of Pulmonary, Allergy and Critical Care Medicine, Department of Medicine, Emory University, Atlanta, Georgia, United States of America; 2 College of Pharmacy, Korea University, Sejong City, Korea; 3 Department of Industrial Engineering, Hanyang University, Seoul, Korea; 4 Division of Endocrinology, Metabolism and Lipids, Department of Medicine, Emory University, Atlanta, Georgia, United States of America; 5 Department of Biomedical Engineering, Georgia Institute of Technology, Atlanta, Georgia, United States of America; 6 Clinical Biomarkers Laboratory, Department of Medicine, Emory University, Atlanta, Georgia, United States of America; National Institute of Agronomic Research, France

## Abstract

The concept of multifractality is currently used to describe self-similar and complex scaling properties observed in numerous biological signals. Fractals are geometric objects or dynamic variations which exhibit some degree of similarity (irregularity) to the original object in a wide range of scales. This approach determines irregularity of biologic signal as an indicator of adaptability, the capability to respond to unpredictable stress, and health. In the present work, we propose the application of multifractal analysis of wavelet-transformed proton nuclear magnetic resonance (^1^H NMR) spectra of plasma to determine nutritional insufficiency. For validation of this method on ^1^H NMR signal of human plasma, standard deviation from classical statistical approach and Hurst exponent (*H*), left slope and partition function from multifractal analysis were extracted from ^1^H NMR spectra to test whether multifractal indices could discriminate healthy subjects from unhealthy, intensive care unit patients. After validation, the multifractal approach was applied to spectra of plasma from a modified crossover study of sulfur amino acid insufficiency and tested for associations with blood lipids. The results showed that standard deviation and *H*, but not left slope, were significantly different for sulfur amino acid sufficiency and insufficiency. Quadratic discriminant analysis of *H*, left slope and the partition function showed 78% overall classification accuracy according to sulfur amino acid status. Triglycerides and apolipoprotein C3 were significantly correlated with a multifractal model containing *H*, left slope, and standard deviation, and cholesterol and high-sensitivity C-reactive protein were significantly correlated to *H*. In conclusion, multifractal analysis of ^1^H NMR spectra provides a new approach to characterize nutritional status.

## Introduction

The concept of fractal dynamics as a means to measure irregularity and unpredictability in biological systems was introduced by Thurner et al. [1]–[3]. Such studies show that irregularity and unpredictability are important features of health and that decreased variability and adaptability are often associated with diseases [4]–[7]. For instance, in the experiments of Ivanov et al. [8], fractal analysis of the human heartbeat showed that a higher Hurst Exponent (*H*), indicative of greater regularity, was associated with disease, while normal heartbeat exhibited more chaotic behavior and lower *H* value while cardiac dynamics in the mathematical and fractal sense is still under investigation [8]–[10]. Based upon this concept, increased regularity could indicate developing sickness, and increased irregularity could indicate recovery. If such concepts are applicable to nutritional insufficiencies, this could provide means to detect nutritional insufficiencies and/or monitor efficacy of nutritional interventions that complement traditional biological and statistical methods. Importantly, such fractal approaches are distinct and can potentially reveal aspects of health and disease that the statistical analyses do not describe.

In a previous fractal analysis of diurnal metabolic variation using wavelet transformed proton nuclear magnetic resonance (^1^H NMR) spectra of human plasma, we found that the monofractal parameter *H* was predictive of the plasma content of cysteine (Cys) [11]. Cys and its dietary precursor methionine (Met), are common sulfur amino acids (SAA) involved in many aspects of human health and cellular function [12]–[15]. We recently used ^1^H NMR spectroscopy of human plasma to study effects of dietary SAA content on macronutrient metabolism [16]. Healthy participants (18–36 y, 5 males and 3 females) were equilibrated for 3 d to adequate SAA, fed chemically defined meals without SAA for 5 d (depletion) and then fed isoenergetic, isonitrogenous meals containing 56 mg⋅kg^−1^⋅d^−1^ SAA for 4.5 d (repletion) [17]–[19]. Results showed effects of SAA intake on lipids, some amino acids, and lactate [18].

**Figure 1:**
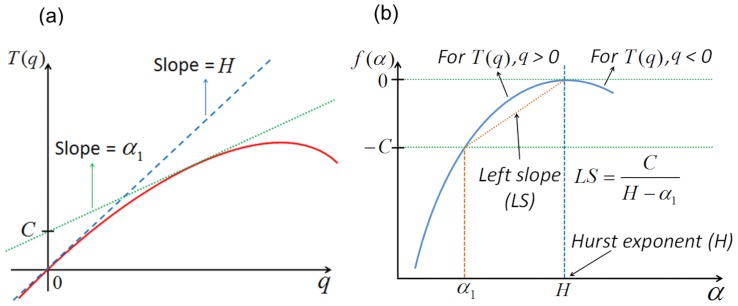
Geometric descriptors for multifractal analysis. (a) The geometric descriptors of the MFS include values of regularity, represented on the horizontal axis and values proportional to the relative frequency of these regularity values, represented on the vertical axis. The maximal regularity is represented by *H* and the overall uncertainty of regularity is provided by the left slope (LS). (b) interpretation of LS with partition function *T*(*q*); LS is obtained by the two slopes (*H* and α_1_) of the two tangent lines; LS is adopted as a measure of deviation from the straight line passing through the origin.

The purpose of present study was to use these ^1^H NMR spectra to test whether fractal analysis can detect nutritional SAA insufficiency. To do this, we used a multifractal approach to improve description of metabolic regularity/irregularity. For reference, the standard deviation (Std) of ^1^H NMR spectra provides means to quantify average variation within spectra. The multifractal analysis decomposes data into subsets characterized by multifractal spectra (MFS) and partition function *T(q)* (Figure 1) with Holder exponent values that quantify local regularity behaviors [20], [21]. The maximum of Holder exponent values is equivalent to the monofractal parameter [22], *H*, capturing important information about MFS (Figure 1A) in a single quantity. The higher value of *H* indicates greater regularity in the spectrum. In contrast, a higher value of left slope (LS), another MFS parameter, indicates the deviation from monofractality, which suggests that the biologic system is more adaptive. The results show that fractal parameters (*H*, LS) along with Std discriminate samples according to SAA sufficiency and suggest that this approach may be useful to detect nutritional insufficiencies and monitor responses to nutritional intervention.

## Materials and Methods

### Human subjects

This study was performed by reanalysis of ^1^H NMR spectral data from previously published studies [17], [18] that were reviewed and approved by the Emory University Investigational Review Board. The blood samples were collected with EDTA, and spectral data were generated for the different projects using procedures and settings with the intent to allow spectral data from different studies to be used for comparative analyses in a cumulative spectral database. Repeat measurements of ^1^H NMR spectra within 24 h showed coefficient of variation to be less than 5%. Spectral data for the primary comparison, SAA insufficiency versus SAA sufficiency, were from a single study so that systematic differences between studies do not affect these comparisons. However, because the other analyses were done weeks apart, there is no way to explicitly exclude unanticipated systematic variations for comparison of the SAA study with the other studies.

Participant characteristics are summarized in Table 1. For the primary comparison of effects of SAA intake, 8 participants in a controlled cross-over study received an isoenergetic, isonitrogenous diet sequentially without and with SAA [18]. Data for 12 critically ill ICU patients were from baseline samples collected upon admission into a double-blind study of albumin treatment [23]. Data for 10 healthy subjects were from a diurnal variation study [24] in which spectra were selected to best match age, BMI and time of day for collection for the ICU patients. In this comparison, the percentage of females in the healthy group (50%) was less than that in the ICU group (75%).

**Table 1:** Individual characteristics.

	Age	Sex	Predisposing Insult	BMI
**ICU**	47	M	Pneumonia	19.7
	49	F	Transfusion	25.5
	54	F	Pneumonia	26.7
	70	F	Sepsis	29.9
	71	M	Sepsis	27.3
	82	F	Sepsis	25.1
	21	F	Sepsis	21.5
	53	F	Pneumonia	36.0
	64	F	Pneumonia	35.0
	67	F	Sepsis	35.4
	76	M	Pneumonia	21.6
	82	F	Sepsis	24.7
**Average of age**	**61.3±17.5**		**Average of BMI**	**27.4±5.6**
**Healthy**	31	F		22.4
	22	F		25.7
	23	M		27.5
	25	M		22.2
	45	F		24.7
	75	M		27.3
	82	F		20.2
	79	M		32.6
	83	F		27.6
	81	M		28.2
**Average of age**	**54.6±8.7**		**Average of BMI**	**25.8±1.1**
**SAA Study**	21	M		20.7
	18	F		22.5
	20	M		22.3
	36	M		20.0
	25	M		21.0
	23	M		23.2
	33	F		26.0
	23	F		24.8
**Average of age**	**24.9±6.4**		**Average of BMI**	**22.6±2.1**

For study of SAA, 8 healthy volunteers without evidence of acute or chronic illness, no current smoking history, and a body mass index (BMI) within the range, 20 to 26, were given an equilibration diet with the RDA for SAA for 3 days. Subjects then received a chemically defined semisynthetic diet with 0 mg/kg SAA per day for 5 days followed by 56 mg⋅kg^−1^⋅d^−1^ SAA for 4.5 days, with a distribution of Met∶Cys of 2∶1. Observations for the 8th subject, however, were not available for all 5 days because of early termination. The protein equivalents were supplied in the form of L-amino acid mixtures (Ajinomoto USA, Teaneck, NJ), providing 1.0 g/kg per day [25], [26]. To compensate for the difference in Met + Cys between the equilibration, 0 and 56 mg⋅kg^−1^⋅d^−1^ SAA diets, the amount of all non-essential amino acids were proportionally changed to maintain a constant dietary nitrogen content while at the same time maintaining them as isoenergetic [25], [27]. Adequate hydration and vitamin, mineral and electrolyte requirements were provided to all subjects to meet or exceed recommended allowances [25], and body weights were determined daily and vital signs were obtained every 8 h.

### ^1^H NMR Spectroscopy

Plasma samples (600 µL) were mixed with 66 µL of deuterium oxide (D_2_O) containing DSS [3-(trimethylsilyl)-1-propanesulfonic acid sodium salt (C_6_H_15_NaO_3_SSi, 1% w/w)], and ^1^H NMR spectra were measured at 600 MHz on a Varian INOVA 600 spectrometer under conditions where stability and reproducibility of the NMR analysis were previously established [16]. Preprocessing of ^1^H NMR spectra containing 11,708 data points included baseline correction with a polynomial regression (NUTS program, Acorn NMR Inc., Livermore, CA), spectral alignment using a beam search algorithm [28] to enhance the computational efficiency of the genetic algorithm [29], elimination of uninformative spectral regions, and normalization relative to the internal standard.

### Wavelet-based Multifractal Analysis

**Figure 2:**
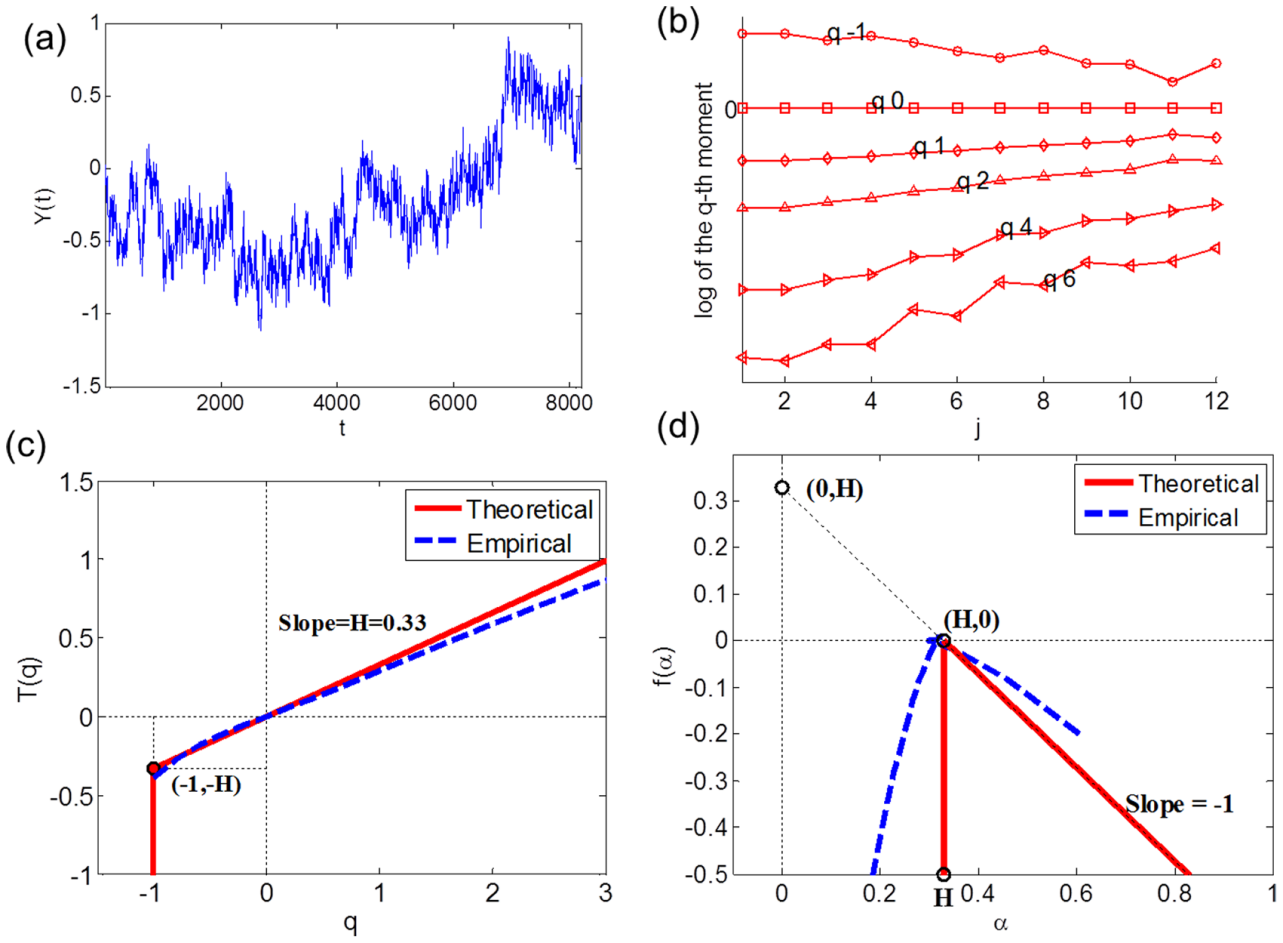
An example of fractional Brownian motions with *H* = 0.33 and its scaling behavior. (a) Signal of fractional Brownian motions with *H* = 0.33 of size 8192, (b) Scaling behavior of the empirical *q* ^th^ order log-average intensity on log-scale *j*, (c) theoretical (red solid line) and empirical (blue dashed line) partition function *T*(*q*), (d) the theoretical (red solid line) and empirical (blue dashed line) multifractal spectra *f*(α) are shown.

Multifractal analysis assesses fractal dimensions of self-similar structures with varying regularities [30] and produces intensity of regularity comprising the MFS [22]. MFS *f*(α) represents a distribution of Holder exponents, or regularity, α. The MFS describes the richness of local regularity in spectra that are more adaptable and complex than can be characterized by a single scaling exponent as used in monofractal analysis. In the present analysis, we used a normalized orthogonal wavelet basis in which the coefficients of discrete wavelet transformation carry information on the local difference near positions on a dyadic scale. Because direct estimation is computationally difficult, we used a partition function [8], *T*(*q*), which was estimated using ordinary least squares (OLS) based on empirical *q* ^th^ moments of the wavelet coefficients with an error term introduced by replacing true moments with empirical ones. In this, *T*(*q*) represents the scaling behavior at exponent *q* between log scales (*j*) of spectra and log-average intensity, capturing high-order dependence in the spectra. For given exponent *q*, the log scales of *j* = 3 and 8, corresponding to 2^−3^ (1/ppm) and 2^−8^ (1/ppm) in terms of ppm frequency, were chosen to compute the slope *T*(*q*) Monofractal spectra display a linear trend of *T*(*q*), proportional to *qH* with *H*, while *T*(*q*) is nonlinear and skewed downward for multifractal spectra (see Figure 1A). For example, a fractional Brownian motion signal *Y(t)* with *H* yields the following partition function *T*(*q*) because the moments of order *q≤−1*of a Gaussian are infinite [22], [31]:
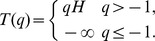

Figure 2A illustrates a signal of *Y*(*t*) with parameter *H* = 0.33 of size 8192. Figure 2B shows the scaling behavior at exponent *q* between log scales (*j*) of the signal and the empirical *q* ^th^ order log-average intensity. Figure 2C shows the theoretical (red solid line) and the empirical (blue dashed line) partition functions for the fractional Brownian motion signal: the empirical one is quite close to the straight line.

Once *T*(*q*) is estimated, the multifractal formalism enables MFS *f*(α) to be calculated by performing a Legendre transformation of the partition function [8]. Monofractal spectra are theoretically reduced to a single point and empirically exhibit narrow parabolicity of *f*(α) around *H*, which means that a high concentration of regularity occurs near *H*. Multifractal spectra, *f*(α), with broad parabolic character, indicates high adaptability and complexity [22], [32]. The location and shape of MFS are summarized without loss of the discriminant information [22] in terms of *H* and left slope (LS) (Figure 1B). For the above fractional Brownian motion example, Figure 2D shows the theoretical (red solid line) and the empirical (blue dashed line) multifractal spectra, of which the maximum is achieved at *H* = 0.33. Analyses generally are limited to the left part of MFS, associated with *T*(*q*), *q>0*, as in Figure 1B, because *T*(*q*) is unstable for negative exponents: that is to say, under the Gaussian assumption, the moments of *q* less than −1 diverge in principle. If both parts of MFS is interested, one can resort to wavelet-leader based multifractal spectra [33]. The wavelet coefficients based method and the descriptors have been successfully used in modeling and classification procedures [34]–[38]. In this study, we focus on *H*, a commonly used regularity measure determined as the apex of the MFS; LS, a measure of deviation from monofractality (Figure 1B). The descriptors naturally include the use of the width of multifractal spectra because the approximate width 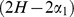 is proportional to 1/LS. Because *H* and LS values focus on the left parts of the MFS and partition function *T*(*q*), we also use the position *q* = 4 in *T*(*q*), i.e., *T*(*4*) as a graphical complement that practically passes the mode.

### Lipid analyses

Triglyceride (mg/dl), high-density lipoprotein (HDL) and low-density lipoprotein (LDL), measured on a Beckman CX7 automatic chemistry analyzer, are from a previous report [39] and expressed as mg/dl.

#### Statistics

The Daubechies 10-tap wavelet filter was used, and all wavelet-related computations were carried out using MATLAB with the Wavelab toolbox and the orthogonal Daubechies filter of 10 vanishing moments [40]. Minitab software (version 15; Minitab, Inc., State College PA) was used for the rest of all statistical analyses. The protocol was designed so that each individual was studied without SAA (depletion) and with 56 mg·kg^−1^·d^−1^ (repletion). Unpaired t test was used to compare partition functions between the healthy and the ICU groups. The significance of the t tests was considered at p≤0.05/3 by Bonferroni correction. A quadratic discriminant analysis was used to separate the group of the SAA depletion and that of the SAA repletion. Analysis of covariance (ANCOVA) of the general linear model (GLM) was used to perform a regression analysis in which continuous (quantitative) variables and categorical (qualitative) variables are involved. Results were considered significant at p≤0.05.

## Results

### Comparison of ^1^H NMR spectra of unhealthy and healthy individuals

As an initial test of the feasibility of application of fractal analysis to ^1^H NMR spectra, we compared spectra from plasma of 12 individuals upon admission to an intensive care unit [23] with 10 healthy subjects participating in a diurnal variation study [16]. The ICU patients included 5 with pneumonia, 6 with sepsis and one following transfusion (Table 1). The unhealthy and healthy subjects had similar mean ages (ICU, 61±18 y; healthy, 55±9 y) and body mass index, BMI (ICU, 27.4±5.6; healthy 25.8±1.1).

**Figure 3:**
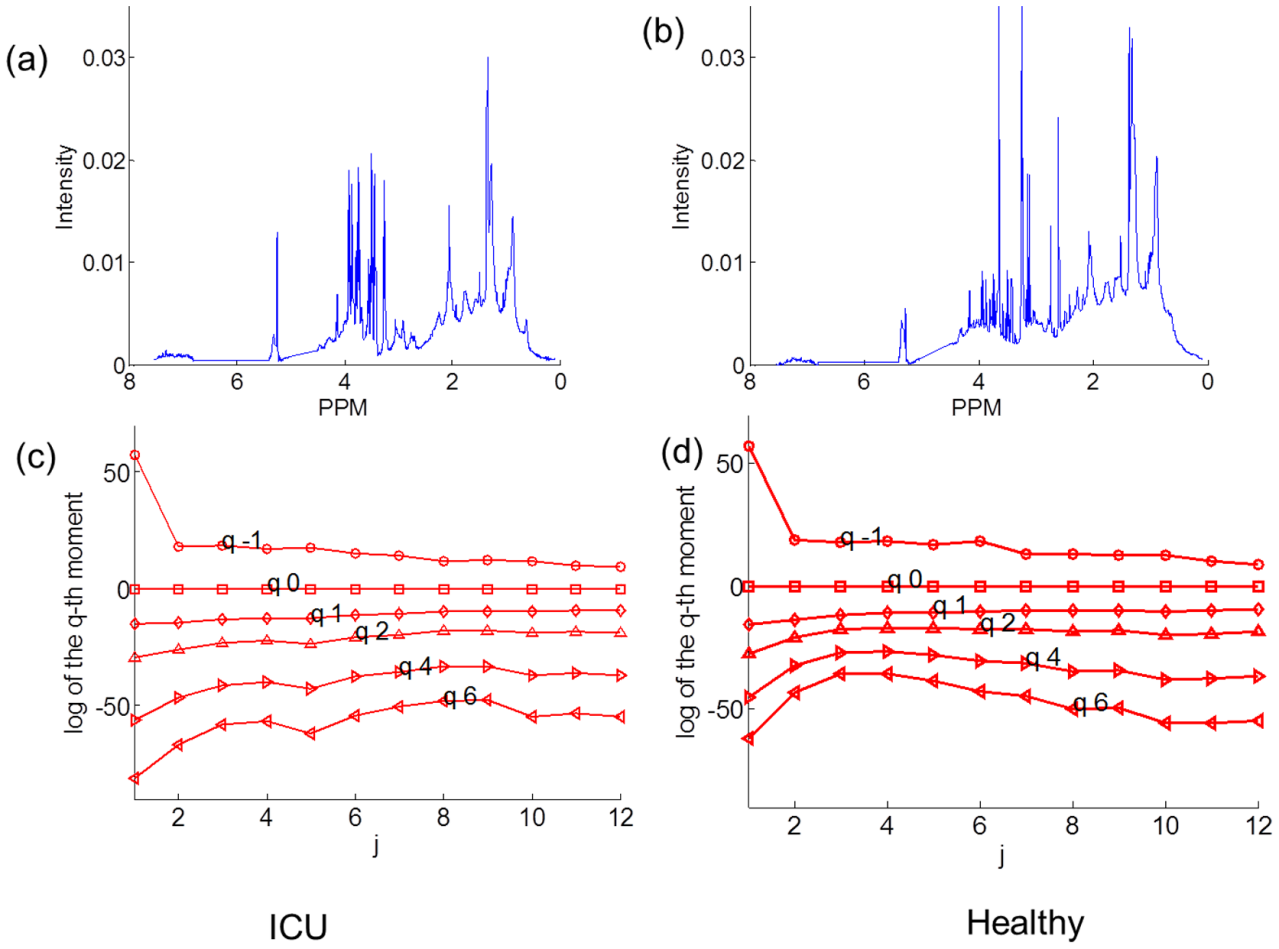
^1^H NMR spectra of an ICU and a healthy individuals and their scaling behavior. ^1^H NMR spectra of an (a) ICU and a (b) healthy individuals are shown. The scaling behaviors of the empirical *q* ^th^ order log-average intensity on log-scale *j* for the (c) ICU and (d) healthy individuals' spectra show that the healthy one produced non-monotonic scaling behavior compared to the ICU one.

**Figure 4:**
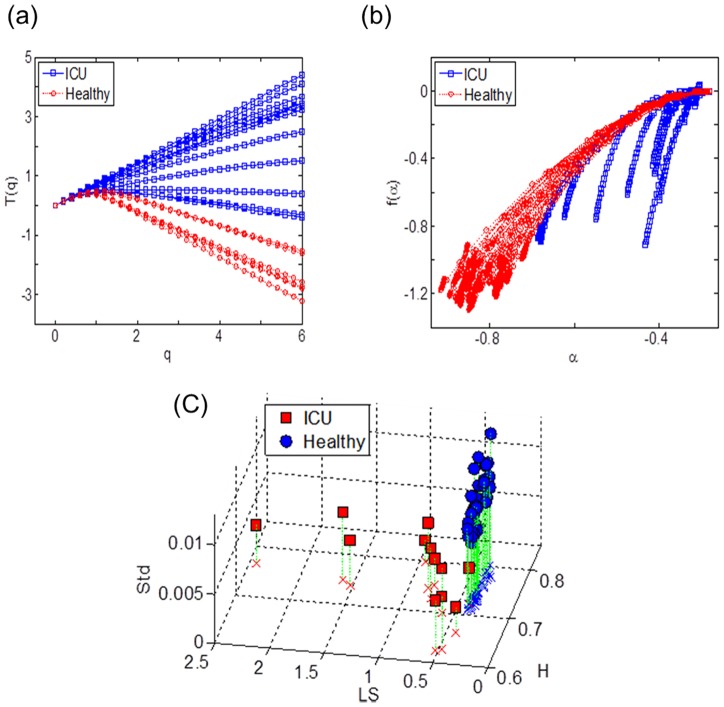
The difference between MFS on ICU patients and healthy subjects. (a) MFSs For the healthy (red) and ICU (blue) individuals, and (b) plots of partition function *T*(*q*) are shown. For the healthy individuals, the MFSs were broad and not steep, and the shapes of *T*(*q*) were skewed downward heavily, which implies that ^1^H NMR spectra of the healthy group is multifractal. (c) classification with *H*, LS, and Std for healthy individuals and ICU individuals. The discriminative characteristics of the healthy group are *H* values around 0.75, small LS values, and high Std values.

NMR spectra for a representative ICU patient (Figure 3A) produced scaling behavior of log-average intensity on log scales (Figure 3C), quite close to that of a fractional Brownian motion as in Figure 2B. On the other hand, NMR spectra for a healthy individual (Figure 3B) produced non-monotonic scaling behavior (Figure 3D) as *q* increases near *j* = 4. The partition function *T*(*q*) for the healthy individuals was skewed downward as the value of exponent q increased (Figure 4A), further indicating that the ^1^H NMR spectra of healthy individuals have characteristics of multifractality. The MFS of the ICU patients (Figure 4B) were steeper than those of the healthy individuals, showing that the spectra of ICU patients were closer to monofractality. The two groups were significantly different in *T*(*4*) (ICU, 1.79±0.31; healthy, −1.15±0.07; *p*<0.001). Hurst exponent values (*H*) of the healthy individuals were densely located (0.747±0.004) as compared to those of ICU (0.718±0.016). The LS values of the healthy individuals (0.372±0.003) were significantly smaller (*p* = 0.008) than those of ICU (0.95±0.18). The Std values of the healthy individuals (0.0092±0.0003) were significantly higher (*p*<0.001) than those of ICU (0.0044±0.0004). The results show that irregularity within ^1^H NMR spectra of plasma is associated with health status, providing support for the hypothesis that multifractal analysis of plasma ^1^H NMR spectra could be used to assess nutritional insufficiency.

### Multifractal analysis of SAA insufficiency

The SAA study was a modified, cross-over design in which participants were given a chemically defined, semi-synthetic diet that was identical except for the content of SAA. Compared to the ICU study, the participants in this study were younger (25±6 y) and had lower BMI (22.6±2.1) [11], so these analyses were considered independent of the above analyses. A graph of *H*, LS, and Std shows that multifractal descriptors separate spectra according to dietary SAA content. Regression analysis showed that *H* (−7.771, *p*<0.001), Std (−216.82, *p*<0.001), and the between-subject variation (*p* = 0.002) were significant in explaining SAA insufficiency. The results show that regularity of plasma ^1^H NMR spectra increases with SAA insufficiency in the participants, suggesting that multifractal analysis of ^1^H NMR spectroscopy of plasma could be used to evaluate aspects of nutritional insufficiency.

**Figure 5:**
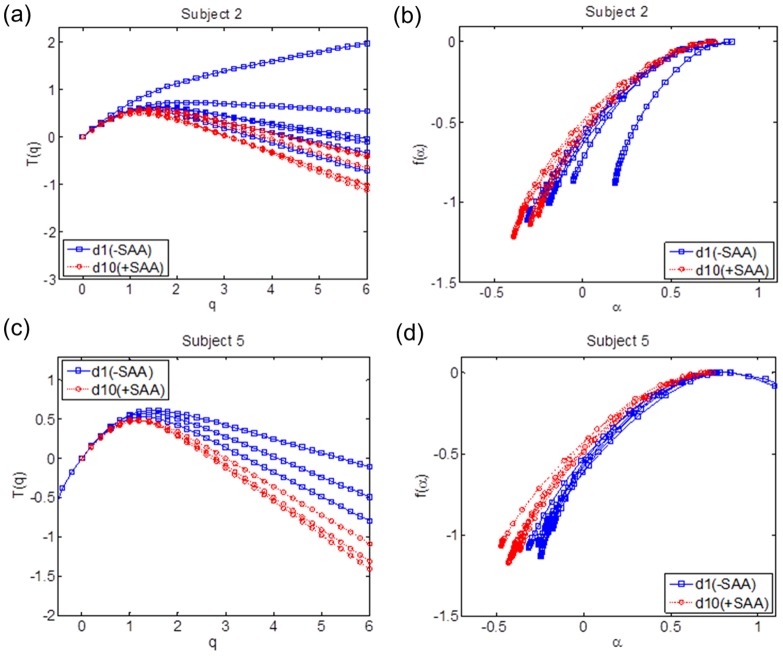
MFS and Partition function on SAA deficiency. MFSs and partition function *T*(*q*) plots for subjects 2 and 5 are shown with d1 of the SAA depletion in red and d10 of the SAA repletion are shown; the numbered markers represent the measurement hours. The effect of the SAA repletion caused MFSs to be broad and partition function *T*(*q*) shapes to be skewed downward.

**Figure 6:**
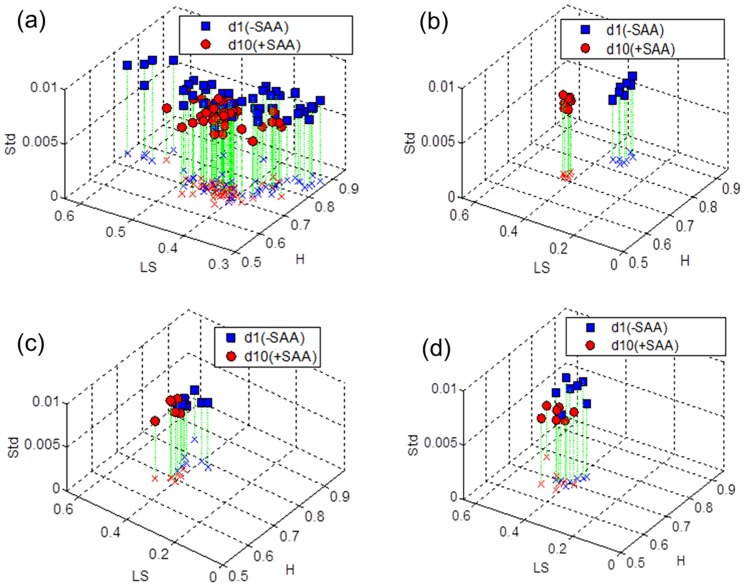
The classification of groups with SAA and without SAA. 3D plots of *H*, LS, and Std for (a) all 7 subjects, (b) subject 1, (c) subject 5, and (d) subject 7 are shown. For all 7 subjects, the three descriptors separated the two groups with 77.5% of correct classification, while the three descriptors produced high separation between SAA repletion and depletion for each individual.

Multifractal spectra and partition function *T*(*q*) plots for representative subjects showed the separation according to SAA intake (Figure 5). The shape of MFS during SAA adequate period was broad and the shape of partition function *T*(*q*) was skewed downward as the exponent q increased. Since the downward trend of *T*(*q*) is evidence of multifractality, the data show that the SAA repletion for the group caused the ^1^H NMR spectra to shift toward more multifractal behavior. Graphs of the multifractal descriptors *H*, LS and Std (Figure 6) similarly show that SAA repletion and depletion are highly separable, with better separation for within-subject comparisons than for overall comparisons between all subjects without and with SAA. Quadratic discriminant analyses showed that the three descriptors discriminated SAA repletion and depletion 77.5% collectively (Table 2). Individually, separations according to SAA intake were more than 90% correct, and 4 subjects had 100% separation (Table 2).

**Table 2:** The results of correct proportion from quadratic discriminant analysis.

Target	Correct Proportion	Target	Correct Proportion
All subjects	77.5%	Subject 4	100%
Subject 1	100%	Subject 5	100%
Subject 2	100%	Subject 6	92.9%
Subject 3	91.7%	Subject 7	92.3%

The descriptors precisely separate the SAA insufficiency from the SAA sufficiency for subjects 1, 2, 4 and 5.

### Association between plasma lipid concentrations and multifractal descriptors

Significant changes in plasma lipid parameters (cholesterol, CHOL; triglycerides, TG; high-sensitivity C-reactive protein, hsCRP; apolipoprotein C3, apoC3) occurred in association with SAA intake [18], so we tested whether the multifractal descriptors of the plasma ^1^H NMR spectra also associated with these clinical measurements. Higher values of CHOL, TG, hsCRP, and apoC3 are indicators of increased cardiovascular disease risk including, so we performed a regression analysis with a model of each of the four measurements as response, *H*, LS, and Std as quantitative variables and subject as a qualitative variable. The results are tabulated in Table 3, in which coefficients are given with *p*-values in parentheses and significant variables underlined. *H* values and subjects were significant for all four models, and all four variables were significant for models of hsCRP and apoC3. Positive signs for the significant coefficients indicate that larger *H* (more regularity) and larger LS (more monofractality), were associated with higher plasma lipid parameters, consistent with the concept that high lipid parameters and more regular ^1^H NMR spectra are associated with poorer health status. For each of these models, the values of the coefficient of determination R^2^, a goodness-of-fit measure of the model, were fairly high (Table 3).

**Table 3:** Association of multifractal descriptors with plasma lipid parameters.

Response	*H*	LS	Std	Subject	R^2^ (%)
CHOL	86.77 (0.011)	43.32 (0.175)	1736 (0.154)	(0.000)	74.87
TG	212.8 (0.015)	445.5 (0.000)	14259 (0.000)	(0.000)	52.59
hsCRP	2.482 (0.026)	0.430 (0.679)	53.89 (0.176)	(0.000)	76.45
apoC3	10.05 (0.008)	7.925 (0.027)	392.3 (0.004)	(0.000)	67.69

Coefficients of response for *H*, LS and Std with *p*-values in parentheses, for a regression model of response in which *H*, LS, and Std are quantitative variables and subject is a qualitative variable in SAA deletion-repletion study. The underlined values represent the significance of the model at a 95% confidence level. The last column represents the coefficient of determination R^2^ for the model.

## Discussion

The concept of fractals based on chaos theory initiated by Mandelbrot [41], [42] provides means to describe complex biological systems and disease, including the severity of Parkinson's disease [5], [6], obstructive sleep apnea [43] and sudden cardiac death [8], [44], [45], with simple numeric values. In this study, we show that multifractal analysis is useful to gain information from ^1^H NMR spectra of human plasma. Consistent with previous studies indicating that an unhealthy condition is associated with more regular characteristics, multifractal analysis of ^1^H NMR spectra of ICU patients showed greater regularity than that seen for healthy individuals. In the study of healthy and ICU patients, all of the multifractal indices measured, including *H*, LS, Std and *T*(*4*), supported the usefulness of multifractal analysis of plasma ^1^H NMR spectra to discriminate healthy from unhealthy individuals.

Based upon these observations, we explored the feasibility to use multifractal analysis for nutritional assessment. ^1^H NMR spectra from a previously published study evaluating the effect of consumption of SAA-free food on the plasma metabolic spectra were employed for this purpose [18]. The nutritional study was performed in a clinical research unit with an established protocol using semisynthetic, chemically defined food in which controlled variation of SAA content was verified. The modified cross-over design with repeat measures, and the consistency in sample collection and analysis, minimized experimental and analytic noise. Previous analyses showed that perturbations in lipid metabolism occurred on the first day of SAA-free food while effects on redox states of thiol-containing amino acids under fasting conditions did not become significant until 4 days on the diet [18]. Even after 4 days, however, one would not normally characterize nutritional status as SAA deficient without further health indications. Thus, the model can be described as having imbalanced amino acid nutrition or SAA insufficiency, but not as SAA deficiency. Because there was no dose-dependent aspect to the study, the sensitivity to detect marginal insufficiency could not be evaluated.

Despite these limitations, the results show that multifractal descriptors separate groups according to SAA status (Table 2). Response times varied for different individuals (data not shown), with some individuals showing greater regularity immediately upon consumption of the SAA-free food while others showed some delay. More detailed studies will be needed to discriminate effects of imbalanced dietary amino acid intake and the metabolic consequences of amino acid insufficiency. Despite this limitation of individual variation, the data showing that two multifractal descriptors (*H* and LS) and Std can predict the levels of lipid-related substances such as cholesterol, triglyceride, hsCRP, and apoC3 (Table 3), suggest that application of multifractal analysis could enhance assessment of diet and disease risk.

In summary, the results show that novel nutritional assessment strategies may be possible using a relatively inexpensive and noninvasive ^1^H NMR spectroscopy analysis of plasma coupled to multifractal analysis. This would appear to be especially useful in that automated ^1^H NMR spectroscopy procedures are capable of analyzing up to 50,000 samples per year [46]. Furthermore, if the methodology could be applied to magnetic resonance spectroscopy of humans employing commonly used MRI instrumentation, then nutritional assessment might be possible in conjunction with diagnostic imaging. Long-term development of multifractal analysis with associated databases of multifractal descriptors could support population-based nutritional assessment and also provide a basis to rapidly screen for personalized dietary imbalance, nutritional insufficiency, and disease risk. As a future research direction to enrich multifractal analysis, other multifractal methods such as wavelet leader approaches and detrended fluctuation approaches could be applied to find out other aspects about nutritional assessment including the role of empirical moments of negative orders.
